# Th1-Biased Immunomodulation and In Vivo Antitumor Effect of a Novel Piperine Analogue

**DOI:** 10.3390/ijms19092594

**Published:** 2018-09-01

**Authors:** Jephesson Santos, Monalisa Brito, Rafael Ferreira, Ana Paula Moura, Tatyanna Sousa, Tatianne Batista, Vivianne Mangueira, Fagner Leite, Ryldene Cruz, Giciane Vieira, Bruno Lira, Petrônio Athayde-Filho, Helivaldo Souza, Normando Costa, Robson Veras, José Maria Barbosa-Filho, Hemerson Magalhães, Marianna Sobral

**Affiliations:** 1 Programa de Pós Graduação em Produtos Naturais e Sintéticos Bioativos, Universidade Federal da Paraíba, 58051-970 João Pessoa, Paraíba, Brazil; jephesson@gmail.com (J.S.); monalisa.brito@gmail.com (M.B.); rafaelcarlos@ltf.ufpb.br (R.F.); anapaulagomesm@hotmail.com (A.P.M.); tatyannakelvia@hotmail.com (T.S.); tatiannemota@gmail.com (T.B.); viviannemangueira@gmail.com (V.M.); fagnercarvalho.farm@gmail.com (F.L.); ryldene@hotmail.com (R.C.); robsonveras@ccs.ufpb.br (R.V.); jbarbosa@ltf.ufpb.br (J.M.B.-F.); hemersoniury@gmail.com (H.M.); 2 Departamento de Morfologia, Universidade Federal da Paraíba, 58051-970 João Pessoa, Paraíba, Brazil; giciane.carvalho@uol.com.br; 3 Departamento de Química, Universidade Federal da Paraíba, 58051-970 João Pessoa, Paraíba, Brazil; brunofrlira@hotmail.com (B.L.); athayde-filho@quimica.ufpb.br (P.A.-F.); helivaldog3@gmail.com (H.S.); normandoalex@uol.com.br (N.C.); 4 Departamento de Ciências Farmacêuticas, Universidade Federal da Paraíba, 58051-970 João Pessoa, Paraíba, Brazil

**Keywords:** cancer, piperine analogue, antitumor activity, toxicity

## Abstract

Natural products have an important role as prototypes in the synthesis of new anticancer drugs. Piperine is an alkaloid amide with antitumor activity and significant toxicity. Then, the *N*-(p-nitrophenyl)acetamide piperinoate (HE-02) was synthesized, and tested for toxicological and antitumor effects. The toxicity was evaluated in vitro (on RAW 264.7 cells and mice erythrocytes) and in vivo (acute toxicity in mice). The Ehrlich ascites carcinoma model was used to evaluate the antitumor activity of HE-02 (6.25, 12.5 or 25 mg/kg, intraperitoneally, i.p.), as well as toxicity. HE-02 induced only 5.01% of hemolysis, and reduced the viability of RAW 264.7 cells by 49.75% at 1000 µg/mL. LD_50_ (lethal dose 50%) was estimated at around 2000 mg/kg (i.p.). HE-02 reduced Ehrlich tumor cell viability and peritumoral microvessels density. There was an increase of Th1 helper T lymphocytes cytokine profile levels (IL-1β, TNF-α, IL-12) and a decrease of Th2 cytokine profile (IL-4, IL-10). Moreover, an increase was observed on reactive oxygen species and nitric oxide production. Weak in vivo toxicological effects were recorded. Our data provide evidence that the piperine analogue HE-02 present low toxicity, and its antitumor effect involves modulation of immune system to a cytotoxic Th1 profile.

## 1. Introduction

Cancer is a generic term that refers to a set of diseases characterized by the presence of cells in continuous proliferation with invasion and metastasis properties [1]. It is one of the most common causes of high morbidity and mortality [2] which is an important public health problem worldwide [3]. Tumor cells share several characteristics, including unregulated proliferation, angiogenesis induction, the ability to escape from immunological detection, and tumor-promoting inflammation [4].

Tumor angiogenesis is one of the markers of tumor progression [5]. It is necessary for the adequate supply of oxygen and nutrients, besides favoring the migration of tumor cells over great distances, leading to metastasis [6]. Thus, angiogenesis represents an important therapeutic target against cancer [7].

The tumor microenvironment is the site of tumor development, which includes not only neoplastic cells, but also stromal cells, such as vascular and lymphatic endothelial cells, pericytes, fibroblasts, myofibroblasts, and various bone marrow-derived cells, such as macrophages and neutrophils [8]. In carcinogenesis, the microenvironment surrounding the tumor provides signs of tumor suppression, to maintain the tissue homeostasis architecture essentially controlled. Nevertheless, once tissue homeostasis is lost, this altered microenvironment becomes a potent tumor promoter. Then, once the tumor is formed, it initiates complex inflammatory and immune responses, in which inflammatory cells are recruited in response to signals from that microenvironment [9].

Tumor cells interfere in both innate and adaptive immunity, and induce macrophages and neutrophils to a type 2 differentiation state, in addition to a change in the response profile of Th1 to Th2 lymphocytes [10]. These cells are able to interfere with each step of the antitumor inflammatory response by secreting mediators that block the function of immune effector cells and reprogram these cells to a regulatory profile [11]. M2 macrophages and Th2 lymphocytes together lead to immunosuppression, angiogenesis and tissue remodeling associated with the release of a set of cytokines, such as IL-4 and IL-10 [12]. These collaborative interactions between neoplastic cells and their stroma aggregate into ectopic structures, which are chronically proliferative and often disseminative [8].

On the other hand, reactive oxygen species (ROS) production by neutrophils and macrophages as a mechanism for tumor cell destruction is already well established. The disproportionate increase of ROS in tumor cells can lead to cell cycle arrest, apoptosis and/or senescence. Furthermore, activated macrophages also generate nitric oxide (NO) that reacts with superoxide to produce peroxynitrite radicals, contributing to tumor cell apoptosis [13,14,15]. Actually, NO has dichotomous effects, modulating different events related to cancer including angiogenesis, apoptosis, cell cycle, invasion and metastasis [16].

Non-clinical and clinical research using a variety of cancer therapies continues to grow worldwide with the prospect of new drug discovery, or to reduce toxicity and the development of resistance related to current drugs. In this context, natural products continue to play an important role as prototypes to the synthesis of new anticancer drugs [17,18,19].

The piperine alkaloid amide (1-piperoylpiperidine) was originally isolated from fruits of black pepper (*Piper nigrum* Linn) and long pepper (*Piper longum* Linn) [20]. Literature data have shown anti-tumor activity in vitro [21,22] and in vivo [23] for piperine. However, piperine and analogues have high toxicity in rodents, characterized mainly by hepatotoxicity [24].

In this study, we investigated the toxicological and antitumor effects of a novel piperine analogue, *N*-(p-nitrophenyl)acetamide piperinoate (HE-02), on the Ehrlich ascites carcinoma model and its mechanism of antitumor action, by evaluating angiogenesis and the tumor microenvironment.

## 2. Results

### 2.1. Chemistry

**Scheme 1:**
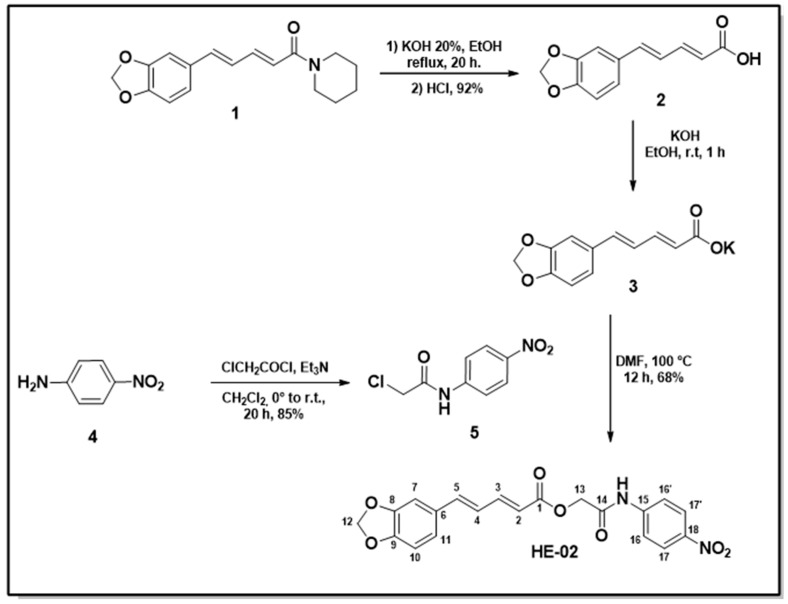
Synthesis of the compound *N*-(p-nitrophenyl)acetamide piperinoate (HE-02). Piperine **1** was obtained by the ethanolic extraction of the ground black pepper (*P. nigrum*) seed in yield ranging from 3–7% [1]. Piperinic acid **2** was obtained from piperine **1** in 92% yield by basic hydrolysis [1]. A neutralization reaction was carried out with piperinic acid **2** in an ethanolic solution of potassium hydroxide, thereby obtaining the potassium piperinoate 3 in high yield. The 2-chloro-*N*-(4-nitrophenyl)acetamide 5 compound was obtained in *N*-acetylation reaction between 4-nitroaniline 4 and 2-chloroacetyl chloride [2]. Reaction between the potassium salt of piperinic acid 3 with 2-chloro-*N*-(4-nitrophenyl)acetamide 5 in *N*,*N*-Dimethylformamide (DMF) provided the formation of *N*-(p-nitrophenyl)acetamide piperinoate HE-02 in yield of 60%.

The synthesis of the compound *N*-(p-nitrophenyl)acetamide piperinoate (HE-02) was carried out according to procedures described in the literature [23,24] and the synthetic steps are outlined in Scheme 1.

The structure of *N*-(p-nitrophenyl)acetamide piperinoate HE-02 was confirmed by IR, ^1^H-NMR and ^13^C-NMR spectroscopic techniques, including 2D Nuclear Magnetic Resonance (NMR) analysis (HMQC). The spectrum in the infrared region showed 3296 cm^-1^ absorption relative to the bond of the N-H group. The subject compound exhibits striking characteristics of ester and amide spectra, which are the absorptions of the strong C=O functional group appearing in 1678 cm^−1^. The C–O–C stretch bands appearing in 1246 cm^−1^ and C–O stretching in 1174 and 1091 cm^−1^. The C=C stretching absorptions of aromatic rings appearing in the 1595 cm^−1^ and 1500 cm^−1^ regions. Thus, in the ^1^H-NMR spectrum revealed two intense singlets, one with an integral for two hydrogens relating to H-13 at δ 4.81 ppm and the other with an integral for two hydrogens referring to H-12 at δ 6.05 ppm. In the region δ 10.74 ppm a signal referring to the hydrogen of the N-H. In the ^13^C-NMR spectra, signals characteristic of C=O ester (C-1) and C=O amide (C-14) at 165.79 and 166.61 ppm respectively. The two-dimensional spectrum of heteronuclear correlations to a (HMQC-^1^J_CH_) distinction between the hydrogen and carbon: δ 6.12 (H-2) with 118.81 (C-2); δ 7.46 (H-3) with 146.22 (C-3); δ 7.06–7.01 (H-4) with 124.57 (C-4); δ 7.06–7.01 (H-5) with 141.14 (C-5); δ 7.24 (H-7) with 105.79 (C-7); δ 6.92 (H-10) with 108.52 (C-10); δ 7.06–7.01 (H-11) with 123.40 (C-11); δ 6.05 (H-12) with 101.42 (C-12); δ 4.81 (H-13) with 62.54 (C-13); δ 7.83 (H-16, 16′) with 118.98 (C-16′, 16′); δ 8.22 (H-17, 17′) with 124.99 (C-17, 17′) ppm.

### 2.2. Evaluation of Cytotoxicity

In the evaluation of cytotoxicity in mice peripheral blood erythrocytes, it was observed that 1000 μg/mL HE-02 induced only 5.01% of hemolysis. Therefore, its HC_50_ has not been determined. On RAW 264.7 cells, HE-02 reduced the viability by 43.10%, 45.22% and 49.75% at the concentrations of 10, 100 and 1000 μg/mL, respectively. Thus, its IC_50_ was not determined.

### 2.3. Assessment of Acute Preclinical Toxicity

On acute preclinical toxicity assay it was shown that the initial 2000 mg/kg treatment of HE-02 did not cause death. Following Organization for Economic Co-operation and Development (OECD) guide 423, the next step was to repeat the previous dose. In this stage, two animals died. A new experiment was carried out with a dose of 300 mg/kg. It was observed that HE-02 did not induce death in any of the experimental animals (Table 1). Then, LD_50_ (lethal dose 50%) value was estimated at around 2000 mg/kg.

No characteristic effects in the central nervous system were observed in any of the doses evaluated at the times evaluated. Regarding the autonomic effects, there was only the presence of diarrhea in the first hour after treatment in the animals treated with the 2000 mg/kg dose.

### 2.4. In Vivo Antitumor Activity

**Figure 1:**
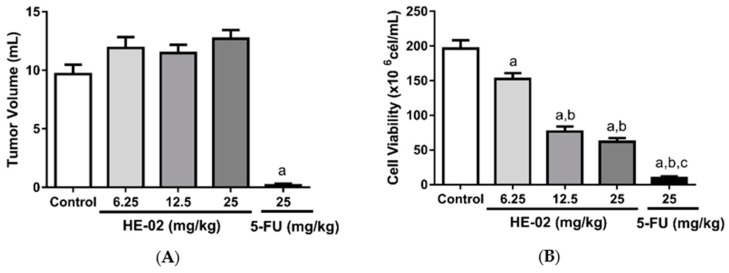
Effect of HE-02 (6.25, 12.5 or 50 mg/kg) on (**A**) Tumoral volume and (**B**) Cell viability. Data presented as mean ± standard error of mean (SEM) of six animals analyzed by analysis of variance (ANOVA) followed by Tukey test. ^a^ *p* < 0.05 compared to tumor control group, ^b^ *p* < 0.05 compared to 6.25 mg/kg dose. ^c^ *p* < 0.05 compared to all HE-02 doses.

HE-02 did not induce any change in the tumor volume parameter. For 5-FU, a significant reduction in volume was observed in comparison to the control group (Figure 1A). HE-02 significantly reduced cell viability in the three doses evaluated, namely: 6.25 mg/kg (152.2 ± 8.25 × 10^6^ cells/mL); 12.5 mg/kg (76.62 ± 7.30 × 10^6^ cells/mL); and 25 mg/kg (61.77 ± 5.33 × 10^6^ cells/mL), as well as 5-FU (9.32 ± 2.76 × 10^6^ cells/mL), when compared to the tumor control group (196.1 ± 12.42 × 10^6^ cells/mL) (Figure 1B).

Considering that 12.5 mg/kg HE-02 was the lowest dose that produced the maximum effect, we choose this dose to study the mechanism of action of HE-02.

### 2.5. Cell Cycle Analysis

Treatment with HE-02 (12.5 mg/kg) did not induce significant change in the different phases of the cell cycle (G0/G1, S and G2/M) when compared to their respective control groups. It was only observed that HE-02 induced a small increase in sub-G1 peak to 23.17%, compared to the control group (14.64%).

**Figure 2:**
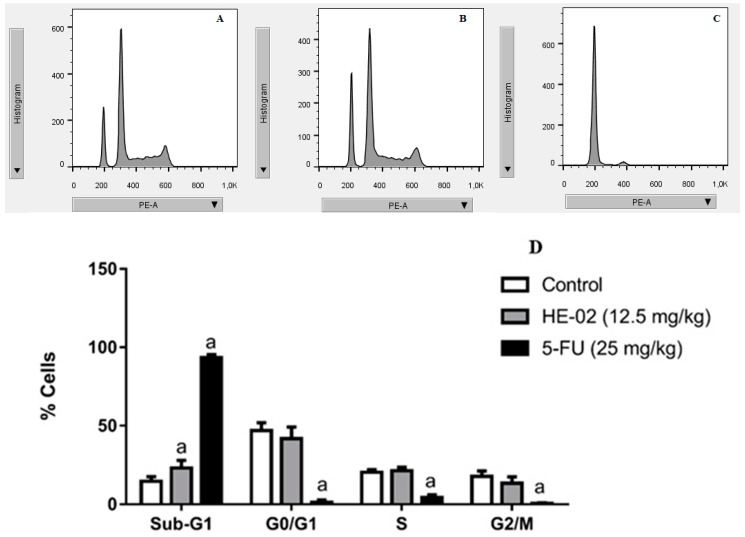
Effects of HE-02 and 5-FU on the cell cycle of Ehrlich ascites carcinoma cells. Representative histograms of ascites carcinoma cells in different phases of the cell cycle after treatment with: (**A**) 12% tween 80 (tumor control); (**B**) HE-02 (12.5 mg/kg); (**C**) 5-FU (25 mg/kg); (**D**) Percentage of Ehrlich ascites carcinoma cells in phases of cell cycle after different treatments. Data presented as mean ± SEM of six animals analyzed by ANOVA followed by Tukey test. ^a^ *p* < 0.05 compared to the tumor control group.

The standard 5-FU drug (25 mg/kg) increased the percentage of cells in the sub-G1 peak to 93.43%, whereas it significantly reduced the percentage of cells in the G0/G1 (1.36%), S (4.40%) and G2/M cells (0.69%), when compared to their respective control groups (G0/G1: 45.70%, S: 20.44%, G2/M: 18.55%) (Figure 2).

### 2.6. Evaluation of Antiangiogenic Effect

**Figure 3:**
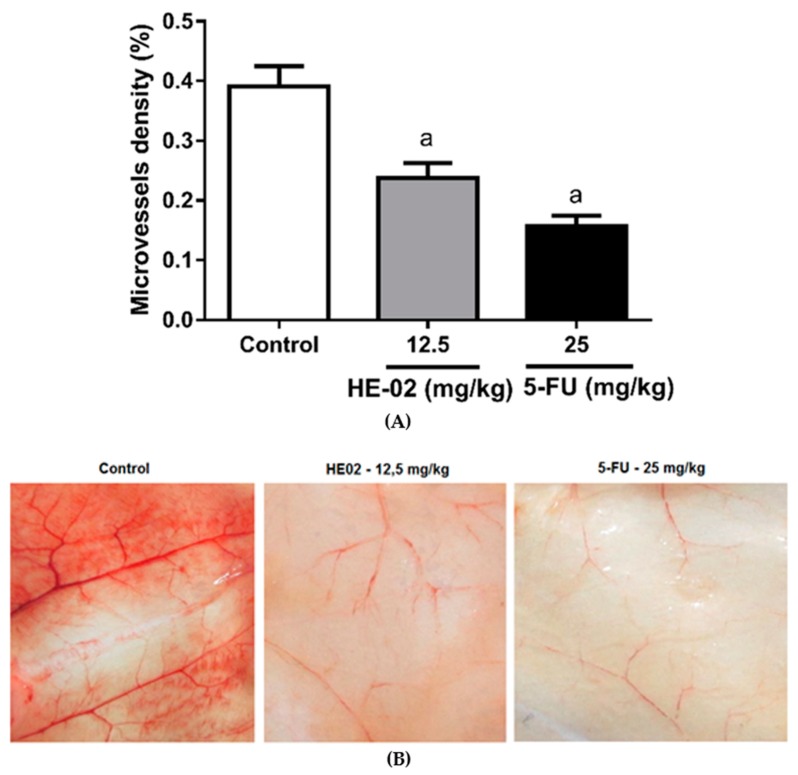
Effects of HE-02 and 5-FU on microvessel density (%) in mice transplanted with Ehrlich tumor, after 9 days treatment. (**A**) Microvessel density (%) represents the blood vessel area per field in selected vascularized areas divided by the whole area. (**B**) Representative images of animals’ peritoneum cut into standard size (1 cm × 1 cm) showing the reduced neovascularization by HE-02 and 5-FU treatments. Data presented as mean ± SEM of six animals analyzed by ANOVA followed by Tukey test. ^a^ *p* < 0.05 compared to tumor control group.

HE-02 (12.5 mg/kg) induced reduction on microvessel density (0.24%), as well as the standard 5-FU drug (0.16%), when compared to the control group (0.39%) (Figure 3).

### 2.7. Quantification of Cytokines in the Peritoneal Lavage

HE-02 (12.5 mg/kg) induced an increase on pro-inflammatory cytokines levels, such as IL-1β (5.42 ± 1.71 pg/mL), TNF-α (672.5 ± 89.91 pg/mL) and IL-12 (472.72 ± 16.70 pg/mL), when compared to their control groups (1.62 ± 0.54 pg/mL, 29.24 ± 5.83 pg/mL and 1.92 ± 0.48 pg/mL, respectively).

Regarding to anti-inflammatory cytokines, there was a decrease on IL-10 (1131 ± 42.52 pg/mL) and IL-4 (400.6 ± 18.19 pg/mL) levels in HE-02-treated animals, when compared to their control groups (1410 ± 37.25 pg/mL and 456.9 ± 16.67 pg/mL, respectively).

**Figure 4:**
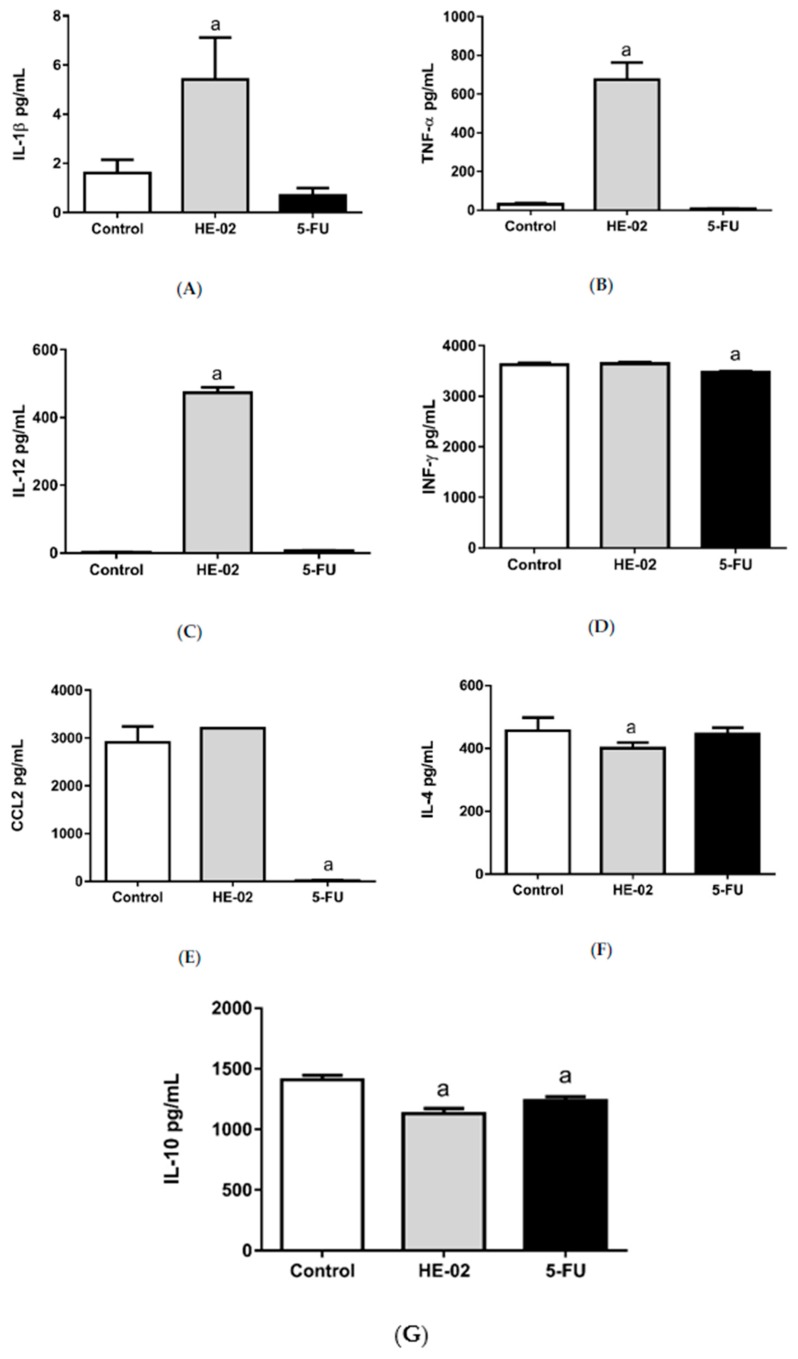
Effect of HE-02 (12.5 mg/kg) and 5-FU (25 mg/kg) on the levels of IL-1β (**A**), TNF-α (**B**), IL-12 (**C**), INF-γ (**D**), CCL2 (**E**), IL-4 (**F**) and IL-10 (**G**) in the peritoneal lavage of Ehrlich ascites carcinoma transplanted mice. Data presented as mean ± SEM of six animals analyzed by ANOVA followed by Tukey test. ^a^ *p* < 0.05 compared to tumor control group.

For 5-FU, reduction was observed on CCL2 (15.81 ± 2.89 pg/mL) and IL-10 (1238 ± 32.05 pg/mL) levels, in comparison to their control groups (2907 ± 196 pg/mL and 1410 ± 37.25 pg/mL, respectively) (Figure 4).

### 2.8. Quantification of Reactive Oxygen Species

**Figure 5:**
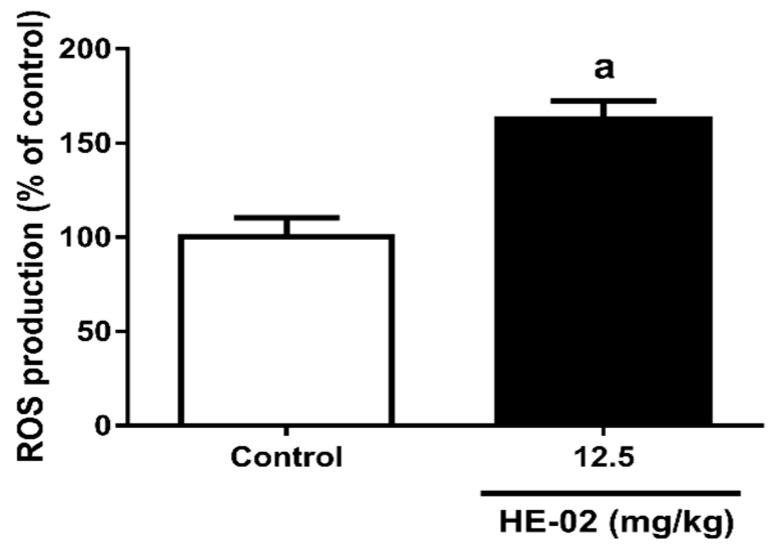
Reactive Oxygen Species (ROS) production (% of control) after treatment with HE-02 (12.5 mg/kg). Data presented as mean ± SEM of six animals analyzed in pairs by Mann–Whitney Test. ^a^ *p* < 0.05 compared to the control group.

It can be observed in Figure 5 that HE-02 (12.5 mg/kg) significantly increased the ROS production (162.9 ± 9.42), compared to the tumor control group (100 ± 10.46).

### 2.9. Quantification of Nitrite Levels

**Figure 6:**
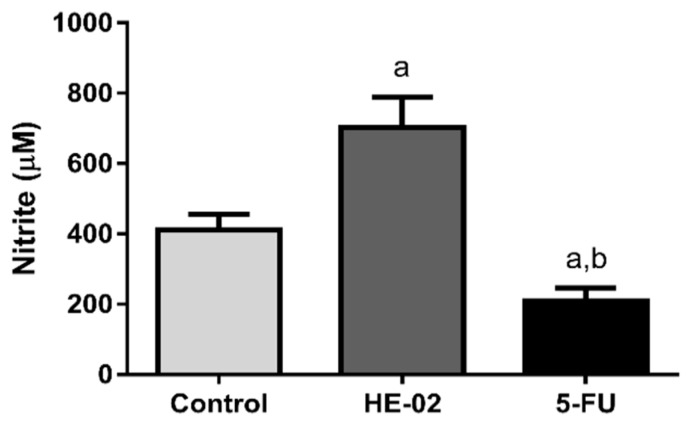
Effect of HE-02 (12.5 mg/kg) and 5-FU (25 mg/kg) on quantification of nitrite in the peritoneal fluid of Ehrlich ascites carcinoma transplanted mice. Data presented as mean ± SEM of six animals analyzed by ANOVA followed by Tukey test. ^a^ *p* < 0.05 compared to tumor control group; ^b^ *p* < 0.05 compared to HE-02 group.

For the group treated with 12.5 mg/kg HE-02, a significant increase in nitrite concentration (703 ± 85.44 μM) was observed in comparison to the control group (410.4 ± 38.66 μM). The 5-FU (25 mg/kg) induced a significant decrease in nitrite concentration (209.1 ± 37.05 μM) (Figure 6).

### 2.10. Toxicity Evaluation for Transplanted Mice

It was observed that HE-02 (12.5 mg/kg) significantly reduced feed and water consumption. As a consequence, there was a reduction of the animal’s weight. Similarly, 5-FU also induced a reduction of the animal’s weight (Table 2).

Regarding the evaluation of biochemical parameters, no significant changes were observed for creatinine, alanine aminotransferase (ALT) and aspartate aminotransferase (AST) parameters. There was only an increase in the blood urea concentration of the animals of treated with HE-02 (Table 3).

On the hematological evaluation, no changes were observed on erythrogram. In relation to the leukogram, HE-02 induced an increase in total leukocytes count, accompanied by a reduction in percentage of lymphocytes, and an increase in the percentage of neutrophils, compared to the tumor control group. For 5-FU a decrease was observed in total leukocytes count along with an increase in the percentage of lymphocytes and a decrease in the percentage of neutrophils (Table 4).

No significant change was observed in kidney, heart and liver index after treatment with HE-02. However, a significant decrease was observed in thymus and spleen index in the HE-02 group, compared to the control group. On the other hand, 5-FU, in addition to the reduction in thymus index, also induced a reduction in liver index and an increase in heart index (Table 5).

**Figure 7:**
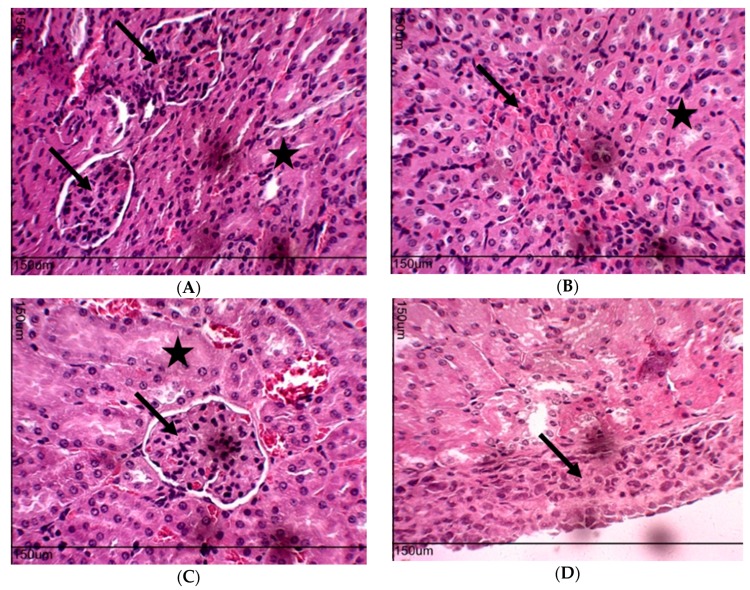

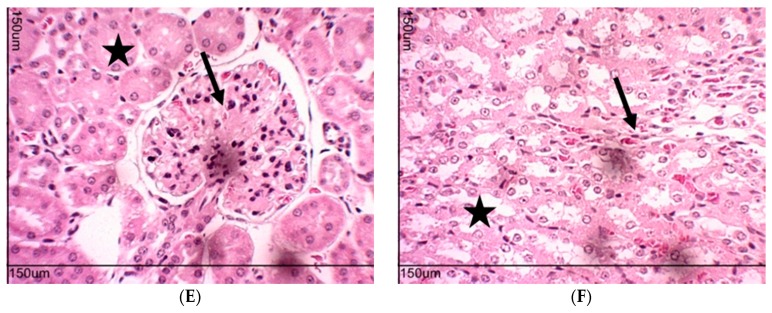
Histopathology of the kidneys of different groups: (**A**) Control, cortical region, renal glomerulus (arrow) and cortical labyrinth (star). (**B**) Control, medullary region, collecting tubules (star), loop of Henle and vessels straight (arrow). (**C**) Group 5-FU, cortical region, glomerulus (arrow) and cortical labyrinth (star). (**D**) Group HE-02 (12.5 mg/kg), subcapsular region, cell infiltrate (arrow). (**E**) Group HE-02 (12.5 mg/kg), cortical region, renal corpuscle (arrow) and cortical labyrinth (star). (**F**) Group HE-02 (12.5 mg/kg), medullary region, loop of Henle and medullar rays (arrow), collecting tubules (star). A, B, C, D, E and F-HE, A.T. ×400.

The histological analyzes of the kidneys of the groups treated with HE-02 (12.5 mg/kg) and 5-FU (25 mg/kg) were within normal histological limits (Figure 7).

The only histopathological finding of HE-02 was the presence of intense cellular infiltrate below the renal capsule (Figure 7D), but characteristics of kidney damage such as glomerulonephritis, tubular necrosis and intratubular protein casts, were not observed.

**Figure 8:**
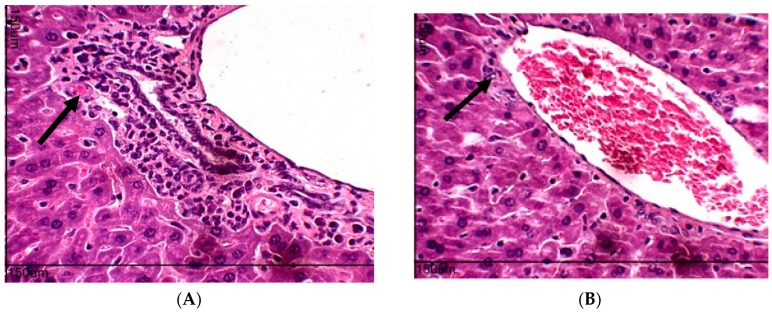

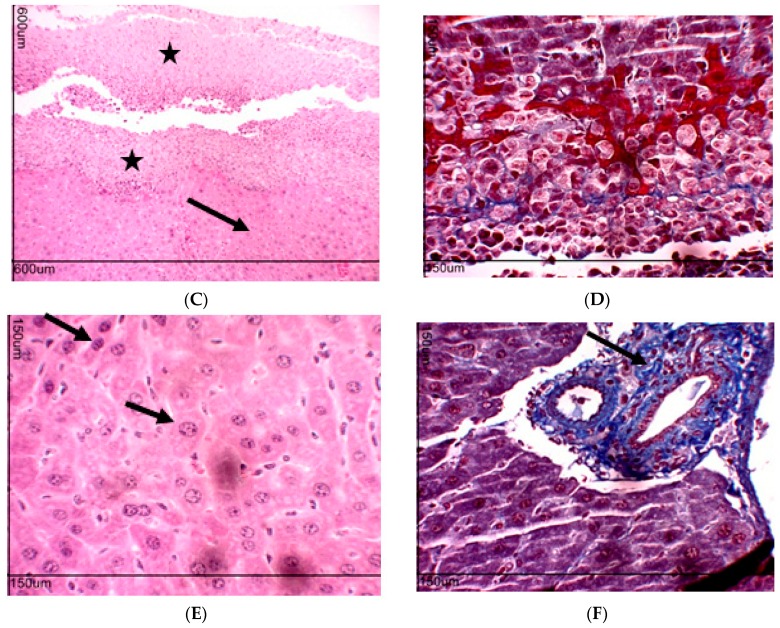
Histopathology of liver of experimental groups. Control group: (**A**) hepatic triad, cellular infiltrate around the hepatic duct; 5-FU: (**B**) cell infiltrate around the branch of the portal vein (arrow); HE-02 (12.5 mg/kg): (**C**) cellular infiltrate in the subcapsular region (star) and hepatocyte plaque (arrow); (**D**) subcapsular cell infiltrate characterized by the presence of macrophages and hepatocytes in apoptosis; (**E**) hepatocytes with varied affinity to both nuclear and cytoplasmic dyes and change in nuclear size (arrow); (**F**) cellular infiltrate in the portal space (arrow). A, B, D, E and F-HE, A.T. ×400. C-HE, A.T. ×100.

The livers removed from tumor control groups presented within normal parameters, although cellular infiltrate observed around the hepatic duct (Figure 8A). Cellular infiltrate was smaller in the 5-FU (Figure 8B) and HE-02 groups (Figure 8C). For the HE-02 group, cellular infiltrate was observed in the subcapsular region of the Glisson capsule (hepatic capsule) (Figure 8C) with presence of mononuclear cells and apoptotic cells forming apoptotic bodies (Figure 8D). Hepatocytes showing changes in nucleus size and dye affinity were observed by alterations in cytoplasmic staining and hepatocyte nucleus (Figure 8E). In addition, cellular infiltrate was observed in the portal space (Figure 8F).

## 3. Discussion

Natural products continue to serve as an important source of anticancer drugs which are used as prototypes for the synthesis of more active and less toxic drugs. In this sense, several piperine analogues have been studied for antitumor and toxicity effects. In the present study, we used piperine as a prototype for the synthesis of a novel analogue, which was tested for toxicity and in vivo antitumor activity.

The cytotoxicity assay in mouse erythrocytes allows us to evaluate the potential of a drug to cause damage to the cell’s plasma membrane, pore formation or the measurement of cell permeability [25]. In addition, erythrocytes are known to be targets of antineoplastic drugs [26]. Therefore, the test represents an important model for evaluating the cytotoxicity of new antitumor drug candidates. This study indicated that HE-02 did not induce damage in the cell membrane, producing low cytotoxicity in erythrocytes of mice. Similarly, piperine was not able to induce hemolysis up to 200 μg/mL [24]. On RAW 264.7 cells, HE-02 induced cytotoxicity from the lowest concentration tested (10 μg/mL), indicating a moderate cytotoxic effect. Nevertheless, until 1000 μg/mL, HE-02 reduced less than 50% of cell viability. For piperine, literature data demonstrate that this substance did not induce cytotoxicity in RAW 264.7 cells at concentrations up to 100 μg/mL [27].

In vivo studies with HE-02 started with the acute preclinical toxicity test in mice, with the objective of determining safe doses to be used in pharmacological tests. Furthermore, a psychopharmacological screening was performed with the objective of qualitatively detecting some important actions of HE-02 on the central nervous system (CNS) and autonomic nervous system (ANS) [28]. Considering the LD_50_ around 2000 mg/kg, as well as the fact that the only observed effect, diarrhea, disappeared after 4 h of treatment with the highest dose tested (2000 mg/kg), it can be inferred that HE-02 has low acute preclinical toxicity in mice, intraperitoneally. Literature data has reported that if the LD_50_ of the test substance is three times more than the minimum effective dose, the substance is considered a good candidate for further studies [29].

To evaluate the in vivo antitumor and toxicological effects of HE-02, we used the Ehrlich ascitic carcinoma model, an undifferentiated and originally hyperdiploid carcinoma, with high transplantation capacity, non-regressive, rapid proliferation, short duration of life, 100% malignant and non-invasive [30]. After nine days of treatment with HE-02, it can be observed that there was no change in tumor volume, suggesting that HE-02 was not able to reverse tumor-induced peritoneal ascites. However, significant inhibition of tumor growth was observed considering the cellular viability parameter, especially at 12.5 and 25 mg/kg. As no significant difference was observed between these doses, 12.5 mg/kg dose was selected for the evaluation of possible mechanisms of action of HE-02. Data from the literature show that *Piper* alkaloids have significant in vivo antitumor activity against the Sarcoma 180 cell line. In these studies, piperine and piplartine showed antitumor activity at 50 and 100 mg/kg dose [24].

Regarding cell cycle analysis, HE-02 induced only slight sub-G1 peaks increase (10% higher than the control group), suggesting that, in vivo, the substance does not act by inducing changes in the cell cycle profile. Literature data show that piperine was able to induce G0/G1 cell cycle arrest in prostate cancer cells of the LNCaP, DU145 and PC-3 lines [31].

Antiangiogenic therapy is a strategy to preventing the growth of new vessels that supply oxygen and nutrients so that tumor cells can proliferate continuously and produce metastasis [32]. Considering that HE-02 could reduce the microvessel density, it may be suggested that this substance exerts its antitumor activity, at least partially, via antiangiogenic mechanisms. Similarly, data from the literature show that piperine has antiangiogenic effects [22].

Several mediators contribute to angiogenesis during tumor development, including cytokines and chemokines [33], reactive oxygen species (ROS) [34] and NO [35]. IL-1β is produced by inflammatory cells and, together with TNF-α, INF-γ and IL-18, activate macrophages and neutrophils for phagocytosis and release of reactive oxygen and nitrogen species [36]. Considering the increase in the concentrations of IL-1β and TNF-α observed after treatment with HE-02, it is possible to infer that this compound modulates the inflammatory response, which may explain the antitumor effect of HE-02 from the activation of macrophages and neutrophils that would be producing ROS and NO to induce cytotoxicity against tumor cells. Piperine also produces immunomodulatory effects. Piperine inhibits transcription factors, such as NF-κB [37], reduces the expression of INF-γ in human peripheral blood mononuclear cells [38], and reduces IL-12 in bone marrow-derived dendritic cells stimulated with LPS [39]. For IL-12, considering its antiangiogenic effect [40], we can suggest that the decrease on peritumoral microvessel density after HE-02 treatment was induced by IL-12 increase. Furthermore, IL-12 is a cytokine that orchestrates the Th1 type immune response, cytotoxic [41].

It is known that the anti-inflammatory cytokines IL-10 and IL-4 are involved in the maintenance of Th2 response profile that, with consequent suppression of the Th1 response, produces a microenvironment favorable to tumor growth [42]. In the present study, HE-02 promoted the reduction of IL-10 and IL-4 levels, suggesting that it promotes the polarization of the Th1 profile.

In addition, HE-02 induced oxidative stress, which suggests that the accumulation of ROS is involved in cytotoxicity against tumor cells. Data from the literature corroborate with these data obtained experimentally for HE-02, since they demonstrate that piperine and analogues induce oxidative stress in tumor cells. For example, piperine, in SK MEL 28 cells, was able to induce DNA damage and cell cycle arrest by inducing ROS production [21].

Nitric oxide has cytotoxic activity, and is produced by natural killer cells, macrophages and Kupffer cells. In addition to direct cytotoxic activity, NO also promotes suppression of DNA synthesis and regulation of apoptosis [16]. The data showed that HE-02 promotes its cytotoxic activity also by the stimulation of NO production. These data corroborate previous data on the quantification of cytokines, mainly IL-1β, TNF-α and IL-12, which together suggest, once again, that the cytotoxic activity of HE-02 is probably due to stimulation of the immune system, polarizing a Th1 profile.

The possible toxic effects of HE-02 on the antitumor treatment were also investigated. Considering that HE-02 induced reduction in water and feed intake as well as, a reduction in the final weight of the animals, it may be suggested that this piperine analogue induces a possible gastrointestinal and/or metabolic toxicity, which needs to be better characterized.

Chemotherapy agents that cause hepatotoxicity produce a predictable pattern of injury where the mechanism is direct or idiosyncratic [43]. However, HE-02 was not able to alter the plasma concentration of the liver enzymes AST and ALT, keeping them within the normal values for the mice of this species, which suggests that HE-02 does not induce hepatic damage in the evaluated conditions.

The biochemical data of AST and ALT are supported by the absence of alteration in liver index, and by the absence of evidence of severe damage to hepatocytes assessed by histological analysis. However, congestion of the branches of the portal vein was observed, which is related to portal pressure. Several factors may alter portal congestion index, ranging from increased portal vein pressure, intrahepatic resistance, increased portal blood flow, and portosystemic collateral circulation. In general, this condition can lead to ascites [44]. This data suggests that the absence of HE-02 effect on intra-abdominal volume, even with the reduction of viability of tumor cells, may be related to ascites induced by hepatic damage, that is, to this portal congestion. Data from the literature have shown liver toxicity for piplartine and piperine, characterized by Kupffer cell hyperplasia, portal tracts and centrolobular venous congestion, infiltrate of inflammatory cells, microvesicular steatosis, intense ballooning degeneration of hepatocytes and sinusoidal hemorrhage [24].

Nephrotoxicity is an inherent adverse effect of certain antitumor agents in several ways [45,46]. The main clinical utility of urea appears to be in the determination in conjunction with creatinine [47,48]. Herein, HE-02 induced only increase in urea plasma concentration. Then, it cannot be confirmed that HE-02 can cause kidney damage. The renal histological study corroborated the biochemical data, and the data of absence of alteration in the kidney index, indicated the preservation of renal structures. Literature data demonstrate that the administration of 100 mg/kg of piperine is able to produce discrete changes in the proximal tubular epithelium, and tubular and proximal hemorrhage [24].

HE-02 induced spleen hypotrophy in the model tested. This result may demonstrate a decrease in the population of periarteriolar cells of the lymphoid sheath, as occurs with the animals treated with piperine. This may represent a reduced B lymphocyte activity, leading to a reduction of antibody titers [49]. In addition, treatment with HE-02 reduced thymus mass, corroborating literature data for piperine [49,50]. Furthermore, piperine causes a decrease in the cell population of the cortical region of the thymus, leading to a suppression of the maturation of T lymphocytes [49]. Based on these findings, it can be suggested that HE-02 can induce a suppression of cellular differentiation of the thymus, thus leading to a decrease in thymic mass.

Changes in leukogram level for HE-02 included leukocytosis, accompanied by reduction of lymphocytes and increase of neutrophils. Leukocytosis and consequently neutrocytosis may be related to the cytotoxic mechanism of HE-02. Lymphopenia, in this case, corroborates with thymus mass data, since these data are complementary to the reduction of lymphocyte maturation in this lymphoid organ. In contrast, piperine led to a reduction in leukocyte concentration [49]. These data demonstrate that piperine analogues may have different hematological toxicity profiles.

In summary, we have obtained a novel piperine analogue which showed antitumor potencial via antiangiogenic and immunomodulatory mechanisms in the sense of inducing a cytotoxic Th1 response. Additionally, we characterized that the replacement of piperidine by an amidoester in HE-02 reduced its toxicity compared to the parent compound. Then, these data support the performance of further preclinical studies with a view to contribute to the discovery of new antitumor drug candidates.

## 4. Materials and Methods

### 4.1. Drugs and Reagents

Propidium iodide, 5-fluorouracil (5-FU), Triton X-100, Tween 80, Tween 20 and cyclophosphamide were purchased from Sigma-Aldrich (St. Louis, MO, USA). Dimethylsulfoxide (DMSO) was purchased from Mallinckrodt CHEMICALS1 (Phillipsburg, NJ, USA). Ketamine-(Ketamin^®^), xylazine hydrochloride-(Anasedan^®^), heparin-(HEPAMAX-S^®^), Buffered phosphate solution (PBS, Sigma-Aldrich), Fetal bovine serum (SBF) (Nutricell^®^), ELISA Kit (eBioscience^®^). Kits for biochemical and hematological analysis were purchased from LABTEST1 (Lagoa Santa, MG, Brazil). The drugs and reagent solutions were prepared immediately before using.

### 4.2. Chemistry

Reagents and solvents used for HE-02 synthesis were analytical grade and purchased from Sigma-Aldrich, Brazil. Thin layer chromatography (TLC) on silica gel plates was used to monitor the reactions progress. The compounds purification was performed by recrystallization in ethanol/water. Melting points were determined on a MQAPF-302 hotplate (Microquímica). Elemental (C, H, and N) analyses were carried out on a Perkin Elmer Elemental Microanalyser. ^1^H- and ^13^C-NMR spectra were obtained in two different machines: a Varian 200 NMR (200 MHz for ^1^H and 50 MHz for ^13^C) and a Varian 500 NMR (500 MHz and 125 MHz ^1^H and ^13^C, respectively). Chemical shifts (δ) were measured in units of parts per million (ppm) and coupling constants (J) in Hertz (Hz). Infrared spectra (IR) were obtained on a Shimadzu model IRPrestige-21 FTIR spectrometer, using KBr pellets.

#### 4.2.1. Sinthesis of *N*-(p-*N*itrophenyl)acetamide Piperinoate (HE-02)

##### Piperine Extraction (**1**)

A total of 150 g black pepper was ground to a fine powder and extracted with 1000 mL 95% ethanol in a Soxhlet extractor for 10 h. The solution was filtered and concentrated in vacuum on a water bath at 60 °C. Alcoholic potassium hydroxide 150 mL 10% was added to the filtrate residue and after a while decanted from the insoluble residue. The alcoholic solution was left overnight, and the precipitate obtained as yellow needles. It was obtained 4.5 g. M.p. 126–128 °C (Lit. [23]: 125–127 °C). IV (KBr, cm^−1^): 3010 (C–H_Ar_); 2930–2860 (C–H); 1634 (C=O); 1580–1490 (C=C_Ar._); 1257 (C–O–C); 930 (C–H_Ar_) [51].

##### Procedure for the Preparation of Piperinic Acid (**2**)

A suspension of 4.40 g (15.44 mmol) of piperine in 44 mL of 20% alcohol KOH solution. The reaction mixture was refluxed and stirred for 20 h. After the completion of the reaction, the mixture was filtered, washed with ethanol and dried. The precipitate formed was solubilized in water and acidified with 10% HCl to pH 3. The precipitate formed with a yellowish color was filtered under reduced pressure, washed with water, dried and recrystallized from ethanol. It was obtained 3.28 g (92.43%). M.p. 217–218 °C (Lit. [23]: 216–217 °C). IV (KBr, cm^-1^): 3250 (OH); 2920 (C–H); 1678 (C=O); 1603–1490 (C=C_Ar._); 1257 (C–O–C); 930 (C–H_Ar_) [51].

##### Procedure for the Preparation of 2-Chloro-*N*-(4-nitrophenyl)acetamide (**5**)

To the mixture of 4-nitroaniline (40 mmol) and Et_3_N (48 mmol, 6.6 mL) in anhydrous CH_2_Cl_2_ (40 mL) at 0 °C, maintained by ice bath, chloroacetyl chloride (48 mmol) was slowly added. The ice bath was removed, and the reaction stayed under agitation for 20 h at room temperature. The reaction mixture was monitored by TLC (hexane/ethyl acetate 1:1). At the end of reaction, the solvent was removed under reduced pressure, the residue was washed with ice water, and the final product was purified by recrystallization with an ethanol/water (1:1) mixture. Yield: 80%; m.p. 188–190 °C. IV (KBr, cm^−1^): 3308, 3273, 3201 (N–H), 3109, 3070 (C–H_Ar._), 2939, 2825 (C-H), 1668 (C=O), 1624, 1506 (C=C_Ar_), 1597, 1570 (NO_2_), 1294, 1255 (C–Cl), 1111, 829 (C–H_Ar._), 748 (N-H), 526 (C–C_Ar_). RMN ^1^H (200 MHz, DMSO): δ 4.36 (d, *J* = 2.3 Hz, 2H, H-2), H-6), 7.85 (dt, *J* = 10.2 e 2.7 Hz, 1H, H-4′) 8,26 (dt, 3H, H-5,5′, H-4′) 10.92 (s, 1H, NH). RMN ^13^C (50 MHz, DMSO): δ 165.62 (C-1), 144.61 (C-3), 142.62 (C-6), 125.07 (C-5,5′), 119.10 (C-4,4′), 43.62 (C-2) [52].

##### Procedure for Obtaining of *N*-(p-Nitrophenyl)acetamide Piperinoate (HE-02)

An equimolar mixture of piperinic acid and potassium hydroxide (5.0 mmol) in absolute ethanol (30 mL) was stirred at room temperature for 1 h. After the solvent was removed under reduced pressure, the residue was treated with 2-chloro-*N*-(4-nitrophenyl)acetamide (5.0 mmol) in DMF (10 mL) at 100 °C for 12 h. The reaction mixture was then cooled, poured on to ice-cooled water and the separated solid was collected, dried and recrystallization from ethanol. Yield: 68%; m.p. 216–217 °C. Anal. Calcd. for C_20_H_16_N_2_O_7_ C (60.61%), H (4.07%), N (7.07%). Found: C (60.56%), H (4.08%), N (7.00%). IV (KBr, cm^−1^): 3296 (N–H), 3093 (C–H_Ar._), 1678 (C=O), 1595, 1500 (C=C_Ar._), 1246 (C–O–C); 1174 (C–O), 852 (C–H_Ar._). RMN ^1^H-(500 MHz, DMSO-*d*_6_, δ): 10.74 (s, 1H, NH); 8.22 (d, 2H, *J* = 9.3 Hz H-17, 17′); 7.83 (d, *J* = 9.4 Hz, 2H, H-16, 16′); 7.46 (ddd, *J* = 15.3; 7.9; 2.4 Hz, 1H, H-3); 7.24 (d, *J* = 1.6 Hz, 1H, H-7); 7.06–7.01 (m, 3H, H-4, 5 and 11); 6.92 (d, *J* = 8.0 Hz, 1H, H-10); 6.12 (d, *J* = 15.2 Hz, 1H, H-2); 6.05 (s, 2H, H-12); 4.81 (s, 2H, H-13). RMN ¹³C-(125 MHz, DMSO-*d*_6_, δ): 166.61 (C-14); 165.79 (C-1); 148.34 (C-9); 148.01 (C-8); 146.22 (C-3); 144.60 (C-15); 142.43 (C-18); 141.15 (C-5); 130.34 (C-6); 124.99 (C-17 e C-17′); 124.58 (C-4); 123.40 (C-11); 118.98 (C-16 and 16′); 118.82 (C-2); 108.53 (C-10); 105.79 (C-7); 101.42 (C-12); 62.49 (C-13).

### 4.3. Animals

Swiss albino mice (*Mus musculus*) were used, weighing between 30 and 33 g, with an approximate age of 60 days, obtained from the Dr. Thomas George Bioterium (Research Institute in Drugs and Medicines/Federal University of Paraíba, Paraíba, Brazil). The animals were grouped in polyethylene cages containing six animals, kept under controlled temperature conditions (21 ± 1 °C), with free access to food (Purina^®^ feed pellets) and water. The animals were kept on a 12 h/12 h off light-dark cycle (lights on at 6:00 a.m.). All procedures were previously approved by the Ethical Committee on the Use of Animals (CEUA)/UFPB. The permission code is 005/2016 approved in June 2017.

### 4.4. Toxicological Assays

#### 4.4.1. Evaluation of Cytotoxicity in Erythrocytes

The erythrocytes were obtained from fresh blood from Swiss mice under anesthesia with ketamine hydrochloride (100 mg/kg, i.m.) and xylazine hydrochloride (16 mg/kg, i.p.) collected from the orbital plexus. The needle was heparinized (sodium heparin) to prevent coagulation. The erythrocytes were resuspended in PBS to obtain the 0.5% (*v/v*) red blood cell suspension. HE-02 was solubilized in DMSO (5%) and prepared in PBS, at twice the desired concentration (1000 μg/mL), and incubated with the erythrocyte suspension in triplicate. Standard and negative control were also used by incubating erythrocytes in a solution of 0.1% Triton X-100 in PBS (2 mL) and DMSO (5%) in PBS (2 mL), respectively. The 96 well plate was maintained under gentle agitation for 60 min, and, the plate was centrifuged for 5 min at 3000 rpm and the supernatant carefully removed. Then, 200 μL/well of Triton X-100 solution (0.1%) was added and the plate was carefully stirred. The amount of hemolysis caused by the Triton X-100 solution (0.1%) was determined spectrophotometrically at 415 nm, and served as a reverse test for the determination of hemolysis percent [53].

#### 4.4.2. Evaluation of Cytotoxicity in RAW 264.7 Macrophage

To evaluate the cytotoxicity of HE-02 against RAW 264.7 cells, we used the 3-(4,5-Dimethylthiazol-2-yl)-2,5-Diphenyltetrazolium Bromide (MTT) assay. For this, the cells were preincubated with vehicle (0.05% DMSO in RPMI 1640) and HE-02 (10–1000 μg/mL). After 4 h of incubation, the MTT reduction assay previously described by Mosmann [54] was performed. The supernatants were removed and the cells were incubated with 200 μL of MTT solution (1 mg/mL) in RPMI-1640 medium for 3 h. The solution was then removed, and the MTT reduced intracellularly to the non-hydrosoluble formazan salt was solubilized with 200 μL of dimethylsulfoxide (DMSO) for 30 min under stirring. The samples were analyzed in a spectrophotometer under wavelength of 570 nm. Results are expressed as percent reduction compared to the control (vehicle) group.

#### 4.4.3. Assessment of Acute Preclinical Toxicity

The acute toxicity test in mice was performed according to the Guideline for Testing of Chemicals 423 of the Organization for Economic Co-operation and Development (OECD) [55]. Mice were divided into different groups (*n* = 3 females/group). Control group received vehicle alone (12% (*v/v*) Tween 80 in saline), and HE-02 was tested in single doses of 300 or 2000 mg/kg, intraperitoneally (i.p.).

For detecting possible behavioral changes suggestive of activity on the Central Nervous System (CNS) or Autonomic Nervous System (ANS), after administration, careful observation was performed to detect general toxic signs in the intervals: 0, 15, 30 and 60 min; after 4 h; and daily for 14 days, using the experimental protocol described by Almeida [28].

### 4.5. Evaluation of In Vivo Antitumor Activity in Ehrlich Ascitic Carcinoma Model

To evaluate the in vivo antitumor activity of HE-02, we used Ehrlich carcinoma cell line which was generously provided by Pharmacology and Toxicology Division, CPQBA, UNICAMP (Paulínia, SP, Brazil). The cells were maintained in the peritoneal cavities of Swiss mice in the Dr. Thomas George Bioterium (Research Institute in Drugs and Medicines/Federal University of Paraíba, Paraíba, Brazil).

Female mice were inoculated with 4.0 × 10^6^ cells/mL of Ehrlich tumor cells (0.5 mL/animal), intraperitoneally (i.p.) [56]. After 24 h, mice were divided into five groups (*n* = 6) and treated with vehicle alone (12% (*v/v* Tween 80 in saline), 5-FU (25 mg/kg) and HE-02 (6.25, 12.5 or 25 mg/kg), for nine consecutive days (i.p.). On the eleventh day, after a 6-h fast, all animals were anesthetized with ketamine hydrochloride (100 mg/kg, i.m.) and xylazine hydrochloride (16 mg/k, i.p.), and peripheral blood samples were collected from the retro-orbital plexus. Then, the animals were euthanized by cervical dislocation, and the volume of ascitic fluid was collected from the peritoneal cavity to determine the tumor volume which was expressed in milliliter (mL). Trypan blue assay was used to evaluate the cell viability.

#### 4.5.1. Cell Cycle Analysis

Cells from ascitic fluid (10^6^ cells) of tumor control, 12.5 mg/kg HE-02 and 25 mg/kg 5-FU groups were centrifuged at 230 *g* for 7 min. Pellet was resuspended in 0.3 mL of hypotonic fluorocromic solution containing RNase (0.5 mg/mL), Triton-X (0.25%) and propidium iodide (PI) (0.25 mg/mL). Then, the DNA content was analyzed by flow cytometry (BD FACSCanto^TM^ II, Woburn, MA, USA) and 10,000 events were acquired. After cell debris removal, a gate was placed then on PE 585/42 nm-W (width) vs. PE 585/42-A (Area) graph to remove doublets on the right of single cell analysis. The gate with single cells was used to analyze cell cycle as a histogram on PE 585/42-A. DIVA 6.0 software was used to analyze the data [57].

#### 4.5.2. Evaluation of Antiangiogenic Effect

To evaluate the antiangiogenic effect of HE-02, animal’s peritoneum of all groups (tumor control, 12.5 mg/kg HE-02 and 25 mg/kg 5-FU) was cut open and the inner lining of the peritoneal cavity was examined and photographed. Microvessel density was calculated as the blood vessel area per field in selected vascularized areas divided by the whole area, using AVSOFT^®^ software [58].

#### 4.5.3. Quantification of Cytokines in the Peritoneal Lavage

The determination of IL-1β, IL-4, IL-10, IL-12, CCL2, TNF-α and IFN-γ cytokine levels was performed using the ascitic fluid collected from the peritoneal cavity of control and treated (12.5 mg/kg HE-02 and 25 mg/kg 5-FU) animals using an ELISA kit following manufacturer’s instructions (BIOSCIENCE, Inc. Science Center Drive, San Diego, CA, USA). The cytokine titers were expressed as the total grams per milliliter (pg/mL) and calculated from standard curves.

#### 4.5.4. Quantification of Reactive Oxygen Species

The method based on the oxidation of the reagent 2,7-dichlorodihydrofluorescein diacetate (DCFH-DA) was used [59]. The analysis was performed using tumor cells obtained from the ascitic fluid collected from the peritoneal cavity of control and treated (12.5 mg/kg HE-02) animals. Then, 2 × 10^5^ cells, 200 μL DCFH-DA solution (0.3 mM) and PBS to 1 mL were incubated in CO_2_ incubator for 30 min at 37 °C. After centrifugation, cells were resuspended in 500 μL PBS and the data acquisition was performed by cytometric flow and 10,000 events were acquired in 530 nm fluorescence and 485 nm excitation length. The quantification of burst or reactive oxygen species (ROS) was estimated by the average intensity of fluorescence.

#### 4.5.5. Quantification of Nitrite Levels

To evaluate NO production, nitrate concentration in the ascitic fluid was measured using Griess reagent [60]. For this, the peritoneal fluid of control and treated (12.5 mg/kg HE-02 and 25 mg/kg 5-FU) was used. Concentrations of nitrite were calculated from a standard curve previously established with known molar concentrations of sodium nitrite. The tests were done in quadruplicate and the values expressed in μM.

### 4.6. Toxicity Evaluation in Transplanted Animals with Ehrlich Tumor Cells

To study HE-02 toxicity, it was evaluated general parameters, such as body weight (at the beginning and end of the treatment), and water and food consumption, evaluated daily for the nine days of the treatment. Organ indices [organ weight (mg)/animal weight (g)] were determined for liver, spleen, thymus, and kidneys. Serum samples were used for biochemical analysis (urea and creatinine levels, and the activities of alanine aminotransferase-ALT and aspartate aminotransferase-AST) while heparinized whole blood was used to determine: hemoglobin (Hb) level, red blood cell (RBC) count, hematocrit (Hct), and the red cell indices mean corpuscular volume (MCV), mean corpuscular hemoglobin (MCH), and mean corpuscular hemoglobin concentration (MCHC) and, total and differential leukocyte counts [61,62]. To histological analysis, portions of the livers and kidneys were fixed in 10% (*v/v*) formaldehyde, cut into sections of 3 mm, and stained with hematoxylin-eosin. Additionally, liver sections were stained with Masson trichrome to examine hepatic fibrosis, [63].

### 4.7. Statistical Analysis

The results are expressed as the mean ± SEM. Statistical analysis of data was performed using analysis of variance (ANOVA), followed by Tukey’s test (*p* < 0.05). For the ROS production evaluation data were analyzed in pairs by Mann–Whitney Test (*p* < 0.05).

## Figures and Tables

**Table 1:** Effects of single-dose (i.p.) administration of the *N*-(p-nitrophenyl)acetamide piperinoate (HE-02) in mice.

Group		D/T ^1^	Effects
Control		0/3	None
2000 mg/kg	Experiment 1	0/3	Diarrhea
	Experiment 2	2/3	None
300 mg/kg	Experiment 3	0/3	None

^1^ D/T—Death/Treaty.

**Table 2:** Water and feed consumption and weight evolution of mice (*n* = 6) subjected to different treatments (9 days).

Groups	Dose (mg/kg)	Water Intake (mL)	Food Intake (g)	Starting Weight (g)	Final Weight (g)
Control		38.82 ± 6.25	31.12 ± 1.19	31.50 ± 0.45	30.71 ± 0.72
HE-02	12.5	32.27 ± 6.84 ^a^	18.15 ± 2.84 ^a^	30.88 ± 0.82	23.22 ± 0.40 ^a^
5-FU	25	38.36 ± 3.93	35.93 ± 6.69 ^a^	32.00 ± 0.62	26.69 ± 0.98 ^a^

Data presented as mean ± SEM analyzed by ANOVA followed by Tukey test. ^a^ *p* < 0.05 compared to tumor control group.

**Table 3:** Effects of HE-02 and 5-FU on biochemical parameters of peripheral blood of mice (*n* = 6) subjected to different treatments (9 days).

Group	Dose (mg/kg)	AST (U/L)	ALT (U/L)	Urea (mg/dL)	Creatinine
Control		181.80 ± 25.28	25.33 ± 3.84	36.83 ± 3.46	0.24 ± 0.1
HE-02	12.5	247.80 ± 45.19	21.75 ± 2.32	55.00 ± 5.30 ^a^	0.31 ± 0.03
5-FU	25	155.20 ± 20.78	22.33 ± 4.55	37.80 ± 1.71	0.26 ± 0.01

Data presented as mean ± SEM analyzed by ANOVA followed by Tukey test. ^a^ *p* < 0.05 compared to tumor control group.

**Table 4:** Effects of HE-02 and 5-FU on hematological parameters of peripheral blood of mice (*n* = 6) subjected to different treatments (9 days).

Group	Control	HE-02 (12.5 mg/kg)	5-FU (25 mg/kg)
Erythrocytes (10^6^/mm^3^)	7.91 ± 0.18	7.31 ± 0.29	7.33 ± 0.22
Hemoglobin (g/dL)	11.59 ± 0.24	11.28 ± 0.31	10.88 ± 0.31
Hematocrit	38.71 ± 1.09	37.17 ± 1.84	34.47 ± 1.39
MCV (fm^3^)	50.19 ± 1.10	50.66 ± 1.42	47.17 ± 1.15
MCH (pg)	14.99 ± 0.40	15.34 ± 0.22	15.02 ± 0.49
MCHC (g/dL)	30.11 ± 0.91	30.63 ± 0.94	31.43 ± 1.29
Total Leukocytes (10^3^/mm^3^)	10.88 ± 0.73	24.70 ± 2.65 ^a^	4.62 ± 0.36 ^a,b^
Lymphocytes (%)	77.00 ± 3.44	31.20 ± 2.13 ^a^	92.33 ± 0.67 ^a,b^
Segmented (%)	19.80 ± 0.49	67.40 ± 1.48 ^a^	5.83 ± 0.48 ^a,b^
Monocytes (%)	1.00 ± 0.24	1.40 ± 0.24	1.20 ± 0.37
Eosinophils (%)	0 ± 0	0 ± 0	0 ± 0

Data presented as mean ± SEM analyzed by ANOVA followed by Tukey test. ^a^ *p* < 0.05 compared to tumor control group, ^b^ *p* < 0.05 compared to 12.5 mg/kg HE-02 group.

**Table 5:** Index of the organs of mice transplanted with Ehrlich ascites carcinoma after treatment with 5% tween 80 (control) solution, H-E02 (12.5 and 25 mg/kg) and 5-FU (25 mg/kg).

Group	Dose (mg/kg)	Heart Index (mg/g)	Liver Index (mg/g)	Kidney Index (mg/g)	Thymus Index (mg/g)	Spleen Index (mg/g)
Control		3.48 ± 0.10	67.23 ± 1.84	10.58 ± 0.24	3.23 ± 0.16	5.38 ± 0.38
HE-02	12.5	3.13 ± 0.12	71.80 ± 1.36	9.75 ± 0.24	2.02 ± 0.22 ^a^	3.22 ± 0.23 ^a^
5-FU	25	4.05 ± 0.08 ^a^	58.96 ± 1.41 ^a^	10.56 ± 0.44	2.08 ± 0.12 ^a^	5.43 ± 0.18

Data presented as mean ± SEM analyzed by ANOVA followed by Tukey test. ^a^ *p* < 0.05 compared to tumor control group.

## Abbreviations

5-FU	5-Fluouracil
ALT	Alanine Aminotransferase
ANOVA	Analysis of Variance
ANS	Autonomic Nervous System
AST	Aspartate Aminotransferase
CCL2	Chemokine (C-C motif) Ligand 2
CEUA	Ethical Committee on the Use of Animals
CH50	Hemolytic Complement
CNS	Central Nervous System
DCFH-DA	2,7-Dichlorodihydrofluorescein Diacetate
DMF	Dimethylformamide
DMSO	Dimethylsufoxide
EAC	Ehrlich Ascitic Carcinoma
ELISA	Enzyme-Linked Immunosorbent Assay
FBS	Fetal Bovine Serum
HCl	Chloride Acid
HE-02	Ethyl 2-oxo-2-(4-nitrophenylamine)-piperinoate
HFS	Hypotonic Fluorochemical Solution
HMQC	Heteronuclear Multiple-Quantum Correlation
IL-10	Interleukin 10
IL-12	Interleukin 12
IL-1β	Interleukin 1 Beta
IL-4	Interleukin 4
INF-γ	Interferon Gamma
LD50	Lethal Dose 50%
MCH	Mean Corpuscular Hemoglobin
MCHC	Mean Corpuscular Hemoglobin Concentration
MCV	Mean Corpuscular Volume
MDA-MB-231	Human Breast Adenocarcinoma Cell Line
MDPI	Multidisciplinary Digital Publishing Institute
MTT	(3-(4,5-Dimethylthiazol-2-yl)-2,5-Diphenyltetrazolium Bromide)
NMR	Nuclear Magnetic Resonance
NO	Oxide Nitric
OECD	Organization for Economic Co-operation and Development
PBS	Phosphate Buffered Saline
ROS	Reactive Oxygen Species
SK MEL 28	Human Melanoma Cell Line
Th1	T helper Cells Type 1
Th2	T helper Cells Type 2
TNF-α	Tumor Necrosis Factor Alpha
UFPB	University Federal of Paraíba
